# Bacterial periplasmic nitrate and trimethylamine-N-oxide respiration coupled to menaquinol-cytochrome *c* reductase (Qcr): Implications for electrogenic reduction of alternative electron acceptors

**DOI:** 10.1038/s41598-018-33857-2

**Published:** 2018-10-19

**Authors:** Nitanshu Garg, Aidan J. Taylor, David J. Kelly

**Affiliations:** Department of Molecular Biology and Biotechnology, The University of Sheffield, Firth Court, Western Bank, Sheffield, S10 2TN UK

## Abstract

The periplasmic reduction of the electron acceptors nitrate (*E*_m_ +420 mV) and trimethylamine-N-oxide (TMAO; *E*_m_ +130 mV) by Nap and Tor reductases is widespread in Gram-negative bacteria and is usually considered to be driven by non-energy conserving quinol dehydrogenases. The *Epsilonproteobacterium* *Campylobacter jejuni* can grow by nitrate and TMAO respiration and it has previously been assumed that these alternative pathways of electron transport are independent of the proton-motive menaquinol-cytochrome *c* reductase complex (QcrABC) that functions in oxygen-linked respiration. Here, we show that a *qcrABC* deletion mutant is completely deficient in oxygen-limited growth on both nitrate and TMAO and is unable to reduce these oxidants with physiological electron donors. As expected, the mutant grows normally on fumarate under oxygen-limited conditions. Thus, the periplasmic Nap and Tor reductases receive their electrons via QcrABC in *C*. *jejuni*, explaining the general absence of NapC and TorC quinol dehydrogenases in *Epsilonproteobacteria*. Moreover, the specific use of menaquinol (*E*_m_ −75 mV) coupled with a Qcr complex to drive reduction of nitrate or TMAO against the proton-motive force allows the process to be electrogenic with a H^+^/2e^−^ ratio of 2. The results have general implications for the role of Qcr complexes in bacterial oxygen-independent respiration and growth.

## Introduction

The cytochrome *bc*_1_ complex (referred to as Complex III in eukaryotic mitochondria) is a highly conserved proton-translocating, quinol-cytochrome *c* reductase (Qcr) that has a major role in oxygen-linked respiration in phylogenetically diverse prokaryotes^1^. The core of the complex consists of the membrane bound Rieske Fe-S protein and a diheam *b* containing cytochrome, combined with a membrane anchored *c*-type cytochrome facing the extracytoplasmic side of the cytoplasmic membrane. The complex functions via an electron bifurcating proton-motive Q-cycle that couples electron transfer from the quinol pool to periplasmic electron acceptors with proton translocation across the cytoplasmic membrane^2^. In one cycle, for every two electrons transferred, four protons are released to the extracytoplasmic side (p-phase) of the membrane and two protons are taken up from the cytoplasmic side (n-phase). The mechanistically similar cytochrome *b*_6  _*f* complex operates in chloroplasts and cyanobacteria, and connects the two photosystems of oxygenic photosynthesis^1^.

In mitochondria, and most lineages of Gram-negative bacteria where it is present, including the *Alpha*- *Beta*- and *Gammaproteobacteria*, the *c-*type cytochrome associated with the Qcr complex is a monohaem protein in the *c*_1_ family, that donates electrons to small soluble periplasmic *c*-type cytochromes which act to shuttle electrons to the terminal cytochrome *c* oxidase. However, uniquely in *Epsilonproteobacteria*, including the pathogens *Campylobacter jejuni* and *Helicobacter pylori* and the rumen bacterium *Wolinella succinogenes*, this cytochrome is a dihaem protein, a member of the cytochrome *c*_4_ family^3^. Phylogenetic and sequence/structure analysis suggests a mutational induced collapse of the dihaem structure during evolution has resulted in the cytochrome *c*_1_ type of molecule^3^. High GC Gram-positive bacteria also have a diheam cytochrome *c* associated with the Rieske/cytochrome *b* core. In these latter bacteria, soluble *c*-type cytochromes are absent; instead the oxidase interacts directly to form a “super-complex” that couples quinol oxidation with oxygen reduction^4–6^.

There are a few important examples where, in addition to its role in cytochrome *c* oxidase linked oxygen respiration, the *bc*_1_ *c*omplex can mediate electron transport to periplasmic reductases. The best studied of these are in denitrifying bacteria, such as *Paracoccus denirificans*, where the nitrous oxide reductase (Nos), nitric oxide reductase (Nor) and the copper- or *cd*_1_-type of nitrite reductases all receive their electrons by a cytochrome *bc*_1_ dependent route, via either cytochrome *c* or a small copper protein acting as an electron shuttle^7–9^. In addition, many Gram-negative bacteria have a periplasmic cytochrome *c* peroxidase that is commonly dependent on the cytochrome *bc*_1_ complex^10^. Genomic studies have revealed that some bacteria have genes for multiple separate Qcr type complexes, although their physiological roles are largely unknown^11^.

The presence of a Rieske/cytochrome *b* core complex containing the atypical diheam cytochrome *c*_4_ in *Epsilonproteobacteria*^3^ is of interest in relation to possible alternative functions of this complex in this group of bacteria. Recently, it was shown that in *W*. *succinogenes*, nitrate respiration via the periplasmic Nap reductase was unexpectedly severely inhibited in a mutant missing the Qcr complex^12^. This conflicts with the current model of nitrate reduction by the active site subunit NapA, which is based on electron transfer from menaquinol through the membrane associated NapGH subunits acting as a quinol dehydrogenase^13,14^.

In this study, we sought to clarify the role of the Qcr complex in the important *Epsilonproteobacterium* *C*. *jejuni*, which is the commonest cause of bacterial food-borne gastroenteritis in many countries^15^. This bacterium has a microaerophilic lifestyle and colonises the caeca of chickens to high levels; it infects humans mainly through consumption of undercooked poultry^16^. In addition to the use of oxygen as a preferred electron acceptor, various strains of *C*. *jejuni* can reduce fumarate, nitrate, nitrite, TMAO/DMSO, tetrathionate and hydrogen peroxide and many of these can support growth under severely oxygen-limited conditions^17–20^. Whether completely anaerobic growth occurs is controversial^17,21^ but due to the presence of a single oxygen-requiring ribonucleotide reductase essential for DNA synthesis^17^, a small amount of oxygen seems necessary for viability.

Although the assembly, composition and functions of the electron transport chains of *C*. *jejuni* have been clarified in recent years^22–24^, it has been assumed, based on models developed in other bacteria, that the major function of the proton-translocating Qcr complex is in oxygen-linked respiration. Here, we provide evidence from mutant studies for a hitherto unappreciated role for QcrABC in both nitrate and TMAO respiration. The results provide a rationale for the puzzling absence of the membrane bound quinol dehydrogenases NapC and TorC, that are essential for periplasmic nitrate and TMAO reduction respectively, in many other bacteria (e.g. *E*. *coli*), that lack a Qcr complex. Moreover, we show that by the use of menaquinol (*E*_m_ −75 mV) as the electron donor to Qcr, the periplasmic reduction of nitrate, TMAO and certain other electron acceptors e.g. tetrathionate, can be an electrogenic process.

## Results

### Isolation and characterisation of a *qcrABC* deletion mutant

**Figure 1:**
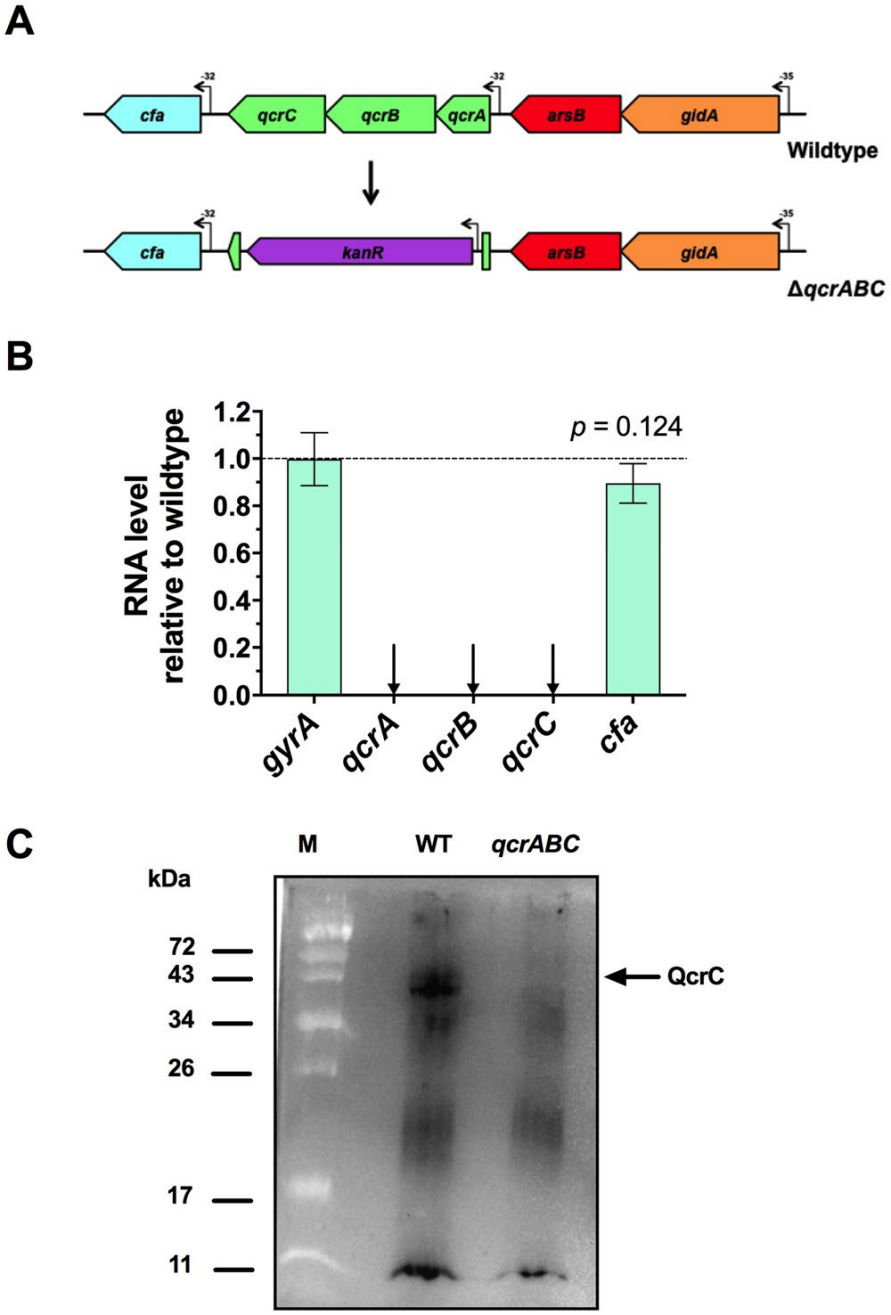
Construction and verification of a *qcrABC* deletion mutant. (**A**) Mutagenesis strategy. The majority of the coding regions of the *qcrA-C* genes were replaced with a kanamycin resistance cassette with its own promoter. The downstream *cfa* gene has its own promoter, but the cassette has no terminator and so should be non-polar on *cfa*. (**B**) RT-PCR to verify expression of *cfa* gene and absence of *qcr* gene transcription in the *qcrABC* mutant. Fold-change in the mutant is shown relative to the wild type strain, using *gyrA* gene as a control. The difference between expression of the *cfa* gene in mutant and wild-type was not significant by t-test. (**C**) shows a comparison of the membrane associated *c*-type cytochromes revealed after a total membrane preparation was subjected to SDS-PAGE and electroblotting to a nitrocellulose membrane, followed by staining for haem-associated peroxidase activity using the chemiluminescence technique described in Methods. 20 μg total protein was run per lane. The Image shown is a cropped version of the full-size blot that can viewed in Supplementary Fig. 1, and was obtained using a ChemiDoc XRS system (BioRad Inc) with an exposure time of 2 min. A band corresponding to the expected size of QcrC is missing in the *qcrABC* deletion mutant.

The genes *cj1186c-cj1184c* are operonic and encode the Rieske FeS subunit, the diheam cytochrome *b* subunit and the dihaem cytochrome *c* subunit, respectively, of the Qcr complex in *C*. *jejuni* (Fig. 1A). Although currently annotated as *petABC* (due to their homology with the *Rhodobacter* **p**hotosynthetic **e**lectron **t**ransport genes encoding a typical cytochrome *bc*_1_ complex), we propose that the *qcrABC* designation be used, as this more accurately reflects their function in this non-photosynthetic bacterium. QcrA and QcrB are similar to many other related homologues, with one transmembrane helix and nine transmembrane helices respectively, while QcrC is predicted to have two transmembrane helices and is significantly larger (41.4 kDa) than the *W*. *succinogenes* homologue (31.7 kDa).

The *qcrABC* genes were deleted from the chromosome of *C*. *jejuni* NCTC 11168 using allelic exchange via a plasmid containing upstream and downstream flanking regions for recombination and where the coding regions were replaced with a non-polar kanamycin resistance cassette with an outward reading promoter^25^ (Fig. 1A). Small colonies that developed on selective plates were shown to have the correct genotype by PCR with flanking primers (Table 1). Despite repeated attempts, we were unable to obtain a complemented strain with the wild-type *qcrABC* genes integrated at a distal locus. However, RT-PCR showed that transcription of the *qcrA*, *qcrB* and *qcrC* genes was absent in the mutant, and that the *cfa* gene, immediately downstream of the *qcrABC* operon, was not significantly altered in expression in the mutant compared to wild-type (Fig. 1B). To further confirm the phenotype of the *qcrABC* mutant, total membranes were prepared by differential centrifugation and subjected to haem blotting as described in Experimental Procedures, to specifically detect *c*-type cytochromes. Figure 1C shows a band of ~42 kDa consistent with size of QcrC (41.4 kDa) is present in wild-type membranes but missing in the *qcrABC* strain (the full size blot can be viewed in Supplementary Fig. 1).

**Table 1:** Primers used in this study.

Name	Sequence 5′− 3′
qcrABC_ISA_F1F	GAGCTCGGTACCCGGGGATCCTCTAGAGTCTGGAGTTTTGCTTTTTAGTTTTG
qcrABC_ISA_F1R	AAGCTGTCAAACATGAGAACCAAGGAGAATGCTTCGTCTACTCTCAGATGTAGC
qcrABC_ISA_F2F	GAATTGTTTTAGTACCTAGCCAAGGTGTGCGCGTTCTGTTTGGTCTAAATTAC
qcrABC_ISA_F2R	AGAATACTCAAGCTTGCATGCCTGCAGGTCATAAAAATCATTACCTATATCATAATGACTTTT
Kan_F	ATTCTCCTTGGTTCTCATGTTTGACAGCTTAT
Kan_R	GCACACCTTGGCTAGGTACTAAAACAATTCAT
qcr_Screen_F	TTAAAATAAGTTTTTTTGCTTTGCT
qcr_Screen_R	AGTTTTTTAAGGGTATGTTCTATTTTGT
RT1186F	GAGAGTAGACGAAGCTTTATG
RT1186R	GTTCTAAGCTCTCCATCTTG
RT1185F	GTAGATTGGCTTGATCAAA
RT1185R	CAAGTGCAGTATCTGGTTT
RT1184F	GCTGTTGAAGATACTACTTTTG
RT1184R	CGATCTTTGCAACATCTAC
RT1183F	GAAACAAACCTGCTAAATTT
RT1183R	CCTTAGCCATTTCATCATA
RTgyrAF	ATGCTCTTTGCAGTAACCAAAAAA
RTgyAR	GGCCGATTTCACGCACTTTA

For RT-PCR primers the designation refers to the relevant gene (e.g. RT1186F is the forward primer for *cj1186c*).

### Comparison of oxygen respiration and microaerobic growth phenotypes of *qcrABC* and *ccoNOQP* mutants: Deletion of *qcrABC* causes accumulation of ROS

**Figure 2:**
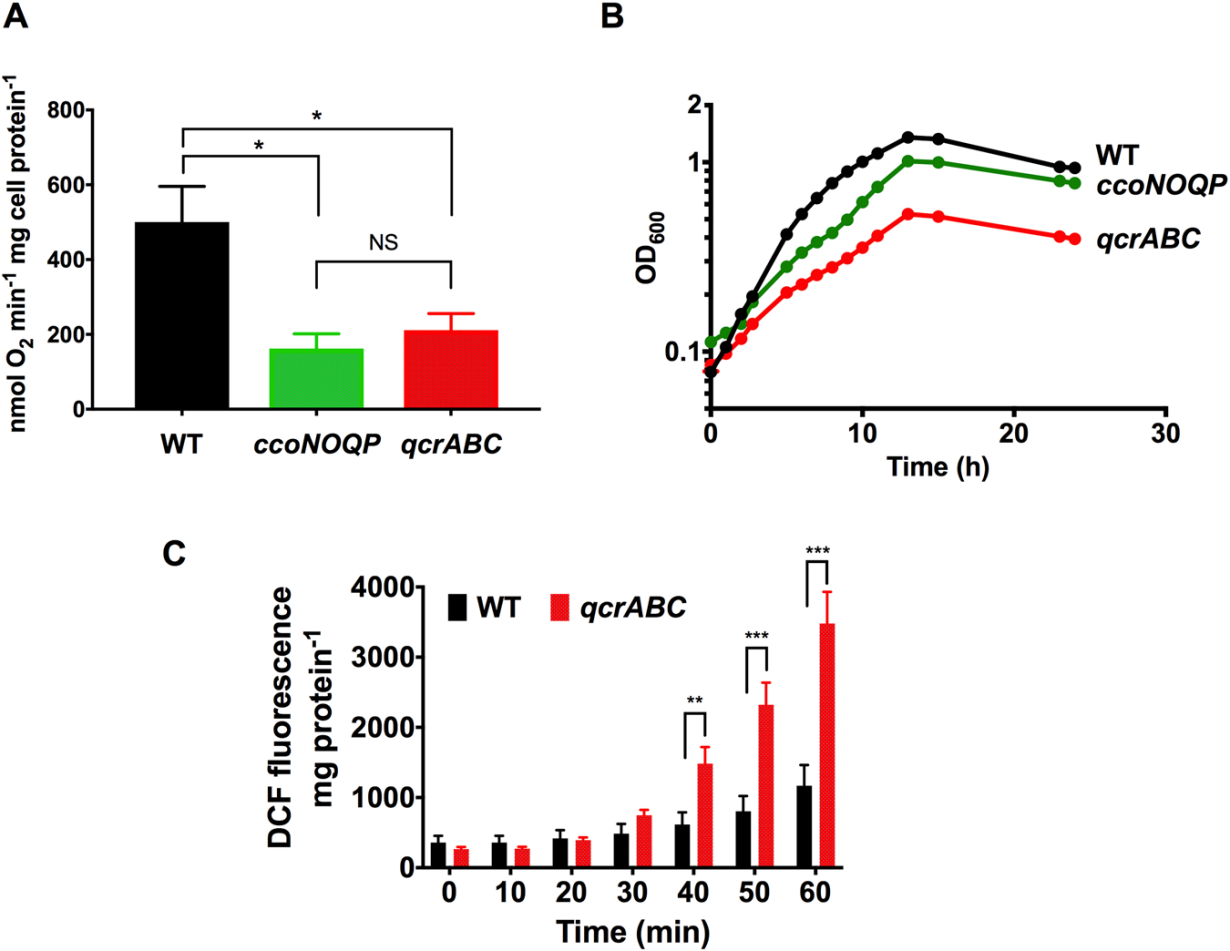
Phenotypic analysis of a *qcrABC* deletion mutant grown under microaerobic conditions. (**A**) shows the specific rate of formate dependent oxygen consumption measured by an oxygen electrode in cell suspensions of the strains indicated. About 68% of the electron flux to oxygen proceeds through the cytochrome c oxidase (CcoNOQP) and, as expected, this is similar in the *qcrABC* mutant, which is the source of electrons for CcoNOQP. The alternative oxidase (CioAB) accounts for the remaining electron flux. The data shown are means and standard deviation of triplicate determinations (*P < 0.05 by one way ANOVA. NS, not significant). (**B**) Growth curves of the strains indicated under microaerobic conditions. The *qcrABC* strain shows a larger reduction in growth rate and cell yield compared to the *ccoNOQP* mutant. The data shown are the means and standard deviations of triplicate growth curves; in most cases the error bars are too small to be seen. (**C**) Accumulation of ROS in microaerobically incubated cells suspensions of wild-type and *qcrABC* mutant strains. The fluorescence emission of 2′,7′ dihydrodichlorofluorescein diacetate (H2DCFDA) added to 10 μM final concentration is shown, normalized to total cell protein. Data are means and standard deviations of triplicate experiments. (**P < 0.01, ***P = 0.001 by multiple t-tests).

Cells of WT, *qcrABC* and a *ccoNOQP* deletion strain^24^, were grown in complex media under standard microaerobic conditions and the specific rate of oxygen consumption in cell suspensions compared, with formate as electron donor. Figure 2A shows that the *qcrABC* and *ccoNOQP* strains had similar rates of formate-linked oxygen respiration but these were 58% and 68% lower respectively compared to the wild-type parent. These results are consistent with electron flux proceeding through the Qcr complex to CcoNOQP, thus giving a similar rate when the cognate genes of either complex are deleted, with the remaining rate being due to oxygen reduction by the alternative Qcr-independent CioAB menaquinol oxidase.

Figure 2B shows growth curves for wild-type, *qcrABC* and *ccoNOQP* strains under microaerobic conditions in complex media with no added exogenous electron acceptors. It is clear that the *qcrABC* strain has a severe growth defect, with a low final cell yield and doubling time of ~6 h in exponential phase compared to ~3.5 h for the wild-type. Interestingly, this is much slower than the *ccoNOQP* deletion mutant (~4.5 h doubling time), which receives electrons from the Qcr complex. We hypothesised that major disruption to the electron transport chain by removal of the Qcr complex might cause an accumulation of reactive oxygen species (ROS), particularly because the two periplasmic cytochrome c peroxidases in *C*. *jejuni* (Cj0020 and Cj0358) are thought to be dependent on the Qcr complex^10,22^. This was tested using the fluorescent ROS sensitive dye 2′,7′ dihydrodichlorofluorescein diacetate (H2DCFDA). We found much higher levels of ROS production in the *qcrABC* strain compared to wild-type when cells were resuspended and incubated in oxygenated buffer (Fig. 2C). Our previous studies using the same method have shown that the same *ccoNOQP* deletion mutant used here does not accumulate ROS above WT levels under these conditions^24^.

### Nitrate and TMAO dependent oxygen-limited growth and respiration requires the QcrABC complex

**Figure 3:**
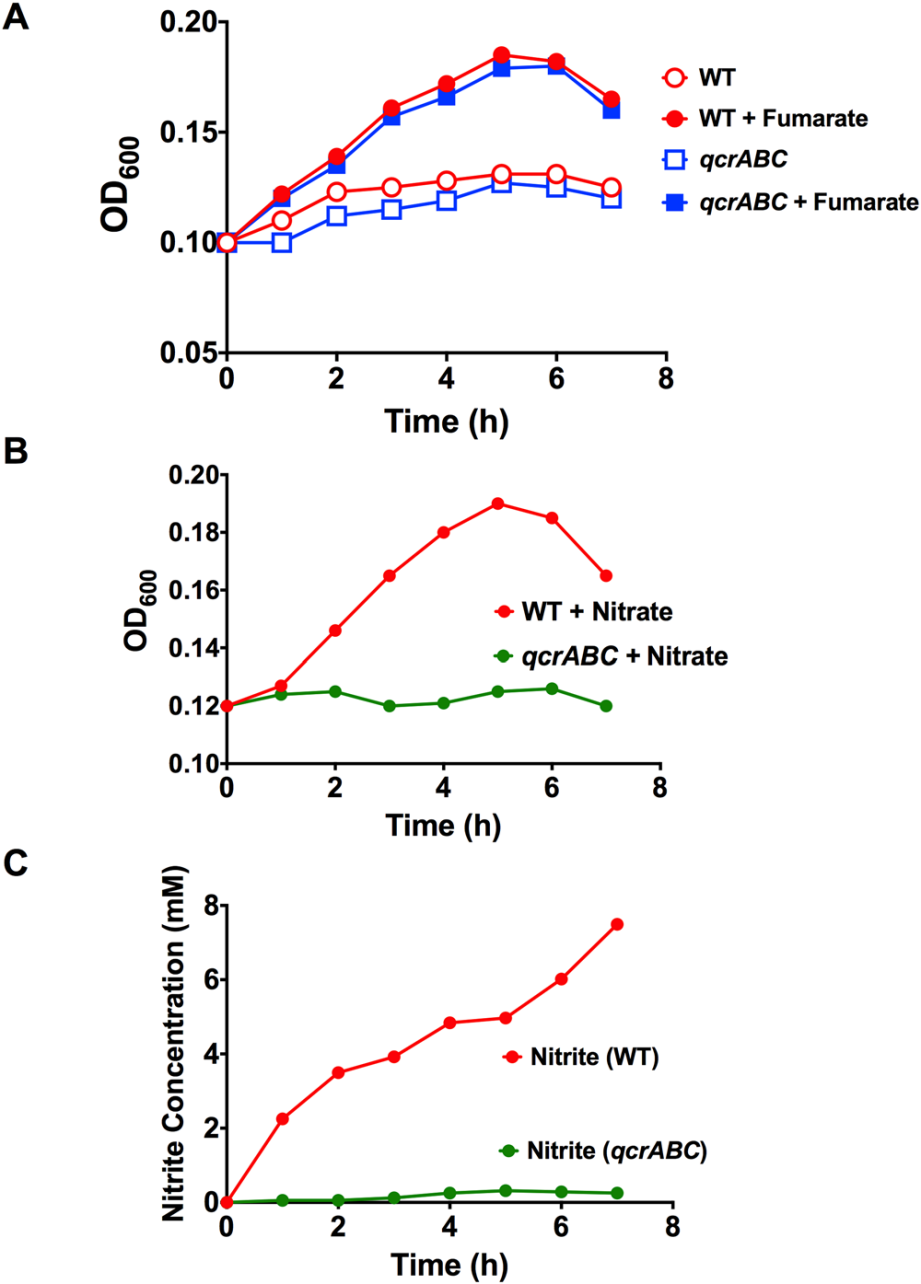
Comparison of growth of wild-type and *qcrABC* strains on fumarate and nitrate under oxygen-limited conditions. (**A**) growth of wild-type (red lines and symbols) and *qcrABC* mutant (blue lines and symbols) in the absence (open symbols) or presence (closed symbols) of 20 mM sodium fumarate. The mutant does not have a growth defect on fumarate, consistent with the two fumarate reductases present in *C*. *jejuni* deriving electrons directly from the (methyl)menaquinol pool. (**B**) growth of wild-type (red line and closed symbols) and *qcrABC* mutant (green line and closed symbols) in the presence of 20 mM sodium nitrate. (**C**) nitrite concentrations were measured in samples taken from the same growth curve as in (**B**) as described in Experimental Procedures. The data shown are single representative growth curves from several that have been performed with independent inocula. In each case similar results were obtained.

Under oxygen-limited conditions in static broth cultures, we have previously shown that growth of *C*. *jejuni* NCTC 11168 is dependent on the addition of a range of alternative electron acceptors including fumarate (*E*_m_ +30 mV), nitrate (*E*_m_ +420 mV) or TMAO (*E*_m_ +130 mV)^17,18^, although growth yields under these conditions are poor compared to microaerobic growth. There are two fumarate reductases present in *C*. *jejuni*, a menaquinol:fumarate reductase (FrdABC complex) and a methylmenaquinol:fumarate reductase (MfrABE complex) and thus electrons pass directly from the (methyl)menaquinol pool to fumarate via these enzymes^19,26^. Figure 3A shows that growth of the *qcrABC* mutant under oxygen-limited conditions with fumarate is identical to that of the parental wild-type strain and that neither strain grows in the absence of fumarate, showing that as expected, fumarate reduction is not dependent on the Qcr complex. This is an important control and the similar growth rate of both mutant and wild-type strains under these conditions, in contrast to the marked aerobic growth defect shown in Fig. 2B, further supports the view that the *qcrABC* mutant experiences significant oxidative stress in the presence of sufficient oxygen.

**Figure 4:**
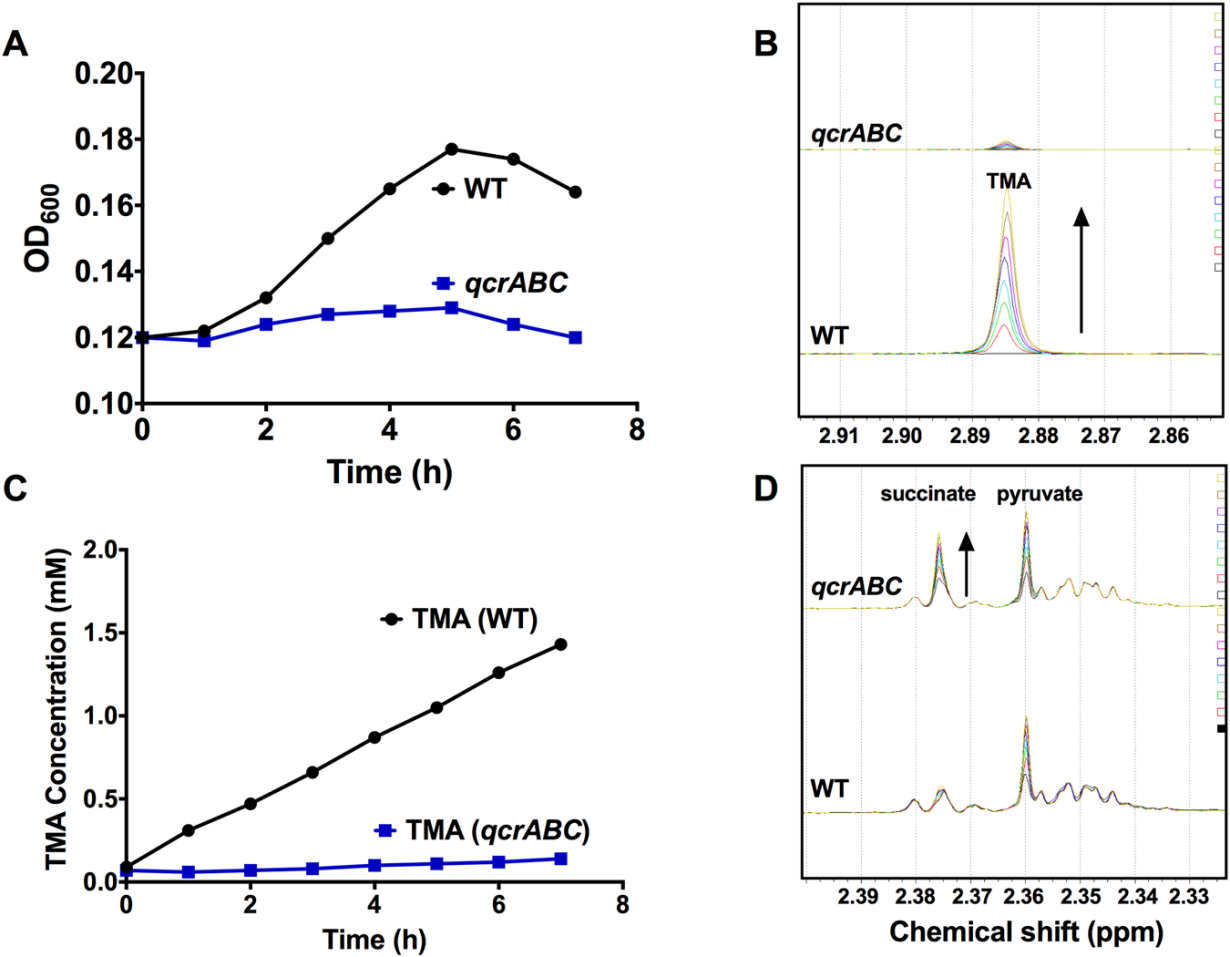
Growth of wild-type and *qcrABC* strains on TMAO under oxygen-limited conditions and measurement of TMA accumulation. (**A**) growth of wild-type (black line and closed symbols) and *qcrABC* mutant (blue line and closed symbols) in the presence of 20 mM TMAO. Representative growth curve of several performed which all gave similar results. (**B**) ^1^H-NMR measurement of TMA accumulation during the growth curve shown in panel A. The region of chemical shift between 2.85 and 2.92 ppm is shown, with the TMA resonance at 2.885 ppm used for quantification. The different coloured spectra are media supernatants from samples taken every hour from time 0 (black spectra) to 7 h (yellow spectra), showing progressive TMA accumulation in the wild-type cells (black arrow) but no significant accumulation in the *qcrABC* mutant. The actual TMA concentrations are plotted in panel (C). Panel (D) shows the region of chemical shift between 2.32 and 2.40 ppm from the same spectra as in panel B, to illustrate the excretion of succinate (black arrow) in the *qcrABC* mutant.

However, similar experiments with nitrate (Fig. 3B) or TMAO (Fig. 4A) as exogenous electron acceptors show that, in contrast to the wild-type, the *qcrABC* mutant is unable to grow with either of these oxidants. Nitrite accumulation from nitrate (Fig. 3C) and TMA accumulation from TMAO (Fig. 4B,C) only occur to any significant extent in wild-type cells and is closely correlated with growth. Taken together, the data indicate that the Qcr complex is required for both nitrate and TMAO-dependent growth and respiration. TMAO reduction was quantified as TMA accumulation at a chemical shift of 2.88–2.89 ppm using ^1^H-NMR spectroscopy of culture supernatants (Fig. 4B,C). During these experiments, we noted a resonance at 2.39 ppm increasing in intensity in the mutant cell supernatants, but much less in the wild-type (Fig. 4D). This peak has a natural abundance ^13^C resonance at 36.8 ppm, which matches succinate; it accumulated to ~1.2 mM after 7 h in the mutant, compared to ~ 0.5 mM in the wild-type. A peak at 2.36 ppm, increased in both WT and mutant (Fig. 4D) and had a natural abundance ^13^C peak at 29.2 ppm, matching pyruvate (~ 2 mM in both strains after 7 h).

### The soluble *c*-type cytochromes CccB (Cj1020) and CccC (Cj0037) are not required for nitrate or TMAO reduction

**Figure 5:**
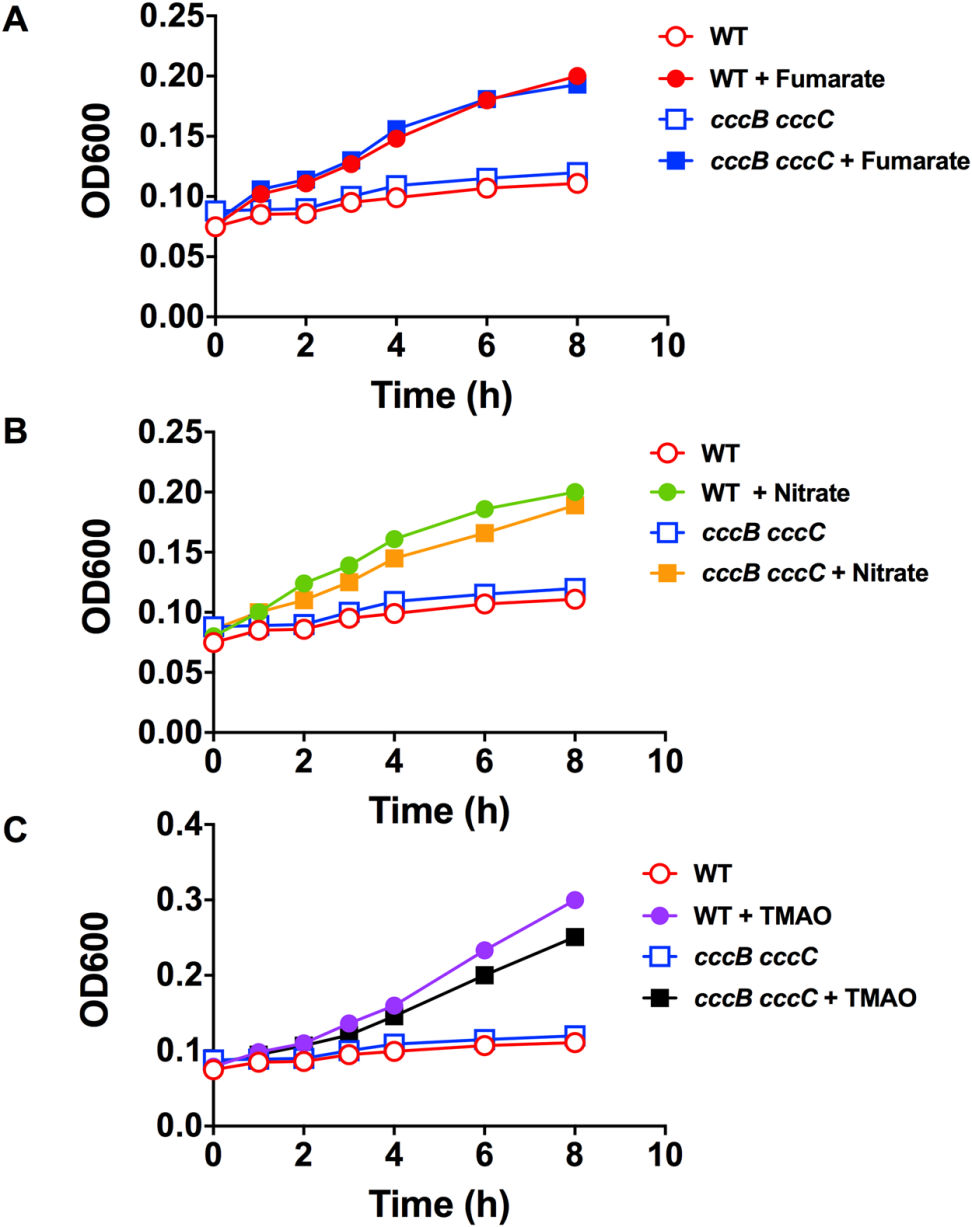
The *c*-type cytochromes CccB (Cj1020) and CccC (Cj0037) are not required for oxygen-limited growth on fumarate, nitrate or TMAO. Panels (A–C) show representative growth curves of wild-type and the *cccB cccC* double mutant in static MHS media alone (open symbols) or in the presence of 20 mM sodium fumarate (closed symbols in A), 20 mM sodium nitrate (closed symbols in B) or 20 mM TMAO (closed symbols in C). The open symbol data are controls for the electron acceptor dependent growth and are the same in each panel.

Electron transfer from the Qcr complex to the Nap or Tor enzymes in the periplasm could occur either directly from QcrC, for example, or via additional periplasmic cytochromes. In *C*. *jejuni*, we previously identified and characterised three major periplasmic *c*-type cytochromes; two monohaem proteins, CccA and CccB, and the dihaem CccC^24^. Deletion mutants in *cccB* or *cccC* show only the loss of the cognate cytochromes, but deletion of *cccA* leads to an unusual pleiotropic phenotype involving the loss of all detectable periplasmic *c*-type cytochromes^24^. Therefore, we could not determine if electron transport to nitrate or TMAO requires CccA by mutant phenotypic studies. However, as Fig. 5 shows, a double *cccB cccC* mutant grows on fumarate, nitrate and TMAO under oxygen-limited conditions just as well as the wild-type, showing that neither of the soluble *c*-type cytochromes CccB or CccC are involved in electron transport to any of these electron acceptors.

## Discussion

**Figure 6:**
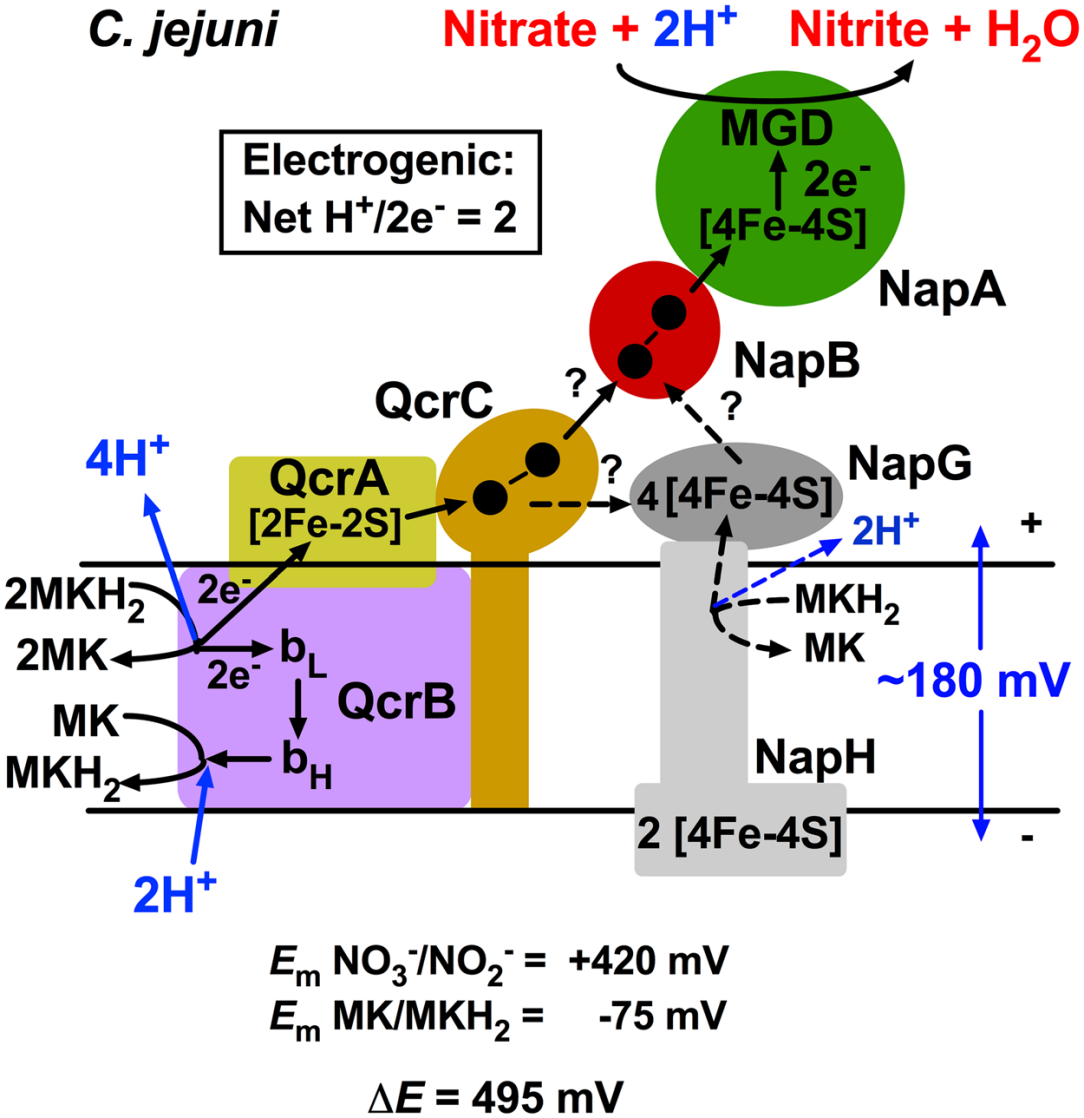
Model for electrogenic nitrate reduction in *C*. *jejuni*. The redox potential span between the MK/MKH_2_ and the nitrate/nitrite couple is large enough to allow for a Qcr dependent electrogenic mechanism, given a typical transmembrane Δ*p* of ~180 mV. The operation of the Qcr complex directly coupled to the NapAB enzyme gives a net transmembrane proton translocation of 2 H^+^ per 2e^−^ transferred from menaquinol. There is uncertainty about the precise route of electron transfer from the Qcr complex to NapB (dashed arrows) but the simplest mechanism would be direct transfer from haem 2 of QcrC to haem 1 of NapB. The role of NapG and NapH, which are known to be essential for nitrate respiration, is unclear. NapG may act in the electron transfer pathway itself or NapGH may act as a quinol dehydrogenase involved in a reductive maturation process e.g. for NapA. Black filled circles in QcrC and NapB represent the haems.

Until very recently, examples of the role of bacterial Qcr complexes in electron transport to a wider range of oxidants than just molecular oxygen was limited to hydrogen peroxide and the reduction of certain nitrogen oxides in denitrifying bacteria. The results presented here clearly show that nitrate and TMAO reduction and growth depend on electron transport through the Qcr complex in *C*. *jejuni*. Our data support recent experiments in the related *Epsilonproteobacterium* *W*. *succinogenes*, which also unexpectedly showed a dependency of nitrate reduction on the Qcr complex^12^. Both bacteria possess a single periplasmic nitrate reductase of the Nap type, with the catalytic NapA subunit receiving electrons via a diheam cytochrome *c* subunit, NapB (Fig. 6). The periplasmic Nap class of nitrate reductases are very widespread in many phylogenetically diverse groups of bacteria and have been thought to be obligately coupled to quinol oxidation through the intermediacy of either the NapC class of tetrahaem quinol dehydrogenases or, in bacteria like *C*. *jejuni* and *W*. *succinogenes*, where NapC is absent, the NapGH proteins (Fig. 6), which have been proposed to form an alternative quinol dehydrogenase module^13,14,27^. In either case, because quinol oxidation (and thus proton release) occurs on the periplasmic side of the membrane, into the same cellular compartment that nitrate reduction (and thus proton consumption) occurs, electron transfer from quinol to nitrate by this mechanism is not energy conserving^14,28^.

The midpoint redox potential of the nitrate/nitrite couple is highly positive (*E*_m_ +420 mV) and in principle nitrate reduction could be driven by either ubiquinol oxidation (*E*_m_ +90 mV) with a Δ*E* of 330 mV, or menaquinol oxidation (*E*_m_ −75 mV) with a Δ*E* of 495 mV. Indeed, in *E*. *coli*, it is thought that NapC catalyses electron transfer from menaquinol to NapB, whereas NapGH is more specific for ubiquinol^29^. For a proton-motive Qcr-dependent mechanism of nitrate reduction, however, the redox potential of the quinol used becomes more critical as a pre-existing proton-motive force of ~180 mV, positive outside, exists across the cytoplasmic membrane, against which the Q-cycle mechanism has to operate. *C*. *jejuni* synthesises menaquinones but not ubiquinone, and so employs the energetically more favourable menaquinol dependent reduction of nitrate which, via the Qcr mechanism, would be electrogenic with a net H^+^/2e ratio of 2 (Fig. 6). Note that recent measurements of Δ*p* in *C. jejuni,* using a permeant cation redistribution method, gave values of ~100–120 mV^30^, but this is an underestimate of the actual value expected for an energy transducing membrane.

Our results, and those of Hein *et al*.^12^ provide a rationale for the absence of NapC in *Epsilonproteobacteria*, but call into question the proposed role of NapGH as a quinol dehydrogenase in these and other bacteria employing a Qcr-complex for nitrate reduction. Nevertheless, from previous mutant studies in *W*. *succinogenes*^31^ it has been established that NapG and NapH are essential for NapA dependent nitrate reduction. In *C*. *jejuni napH* is also essential for nitrate respiration and deletion of *napG* is highly deleterious^18^, with residual growth thought to be due to the ability of the quinol dehydrogenase of the nitrite reductase (NrfH) to donate electrons to NapB^18^. NapG and NapH are possibly involved after electrons leave the Qcr complex, but the route by which electrons might be transferred to them requires investigation. NapG is membrane bound but facing the periplasm, with several FeS redox centres and so direct transfer from the quinol oxidase site in QcrB or the periplasmically located *c*-haems in QcrC are both possible. However, in the light of our current results, we think it more likely that QcrC can donate electrons directly to NapB and that NapGH has some other role (Fig. 6), for example in the reductive maturation of NapA, as has been discussed previously^32^. Irrespective of their precise function in the process, the key bioenergetic consequence of nitrate reduction being dependent on the Qcr complex is that electron transfer from menaquinol to nitrate will be energy conserving. It should be noted that in addition to the Nap-type of nitrate reductase, evidence is also emerging for a role for the Qcr complex in nitrate reduction in *Streptomyces*, which uses the membrane bound Nar-type of enzyme^33^, possibly suggesting direct Qcr-Nar electron transfer in this case.

**Figure 7:**
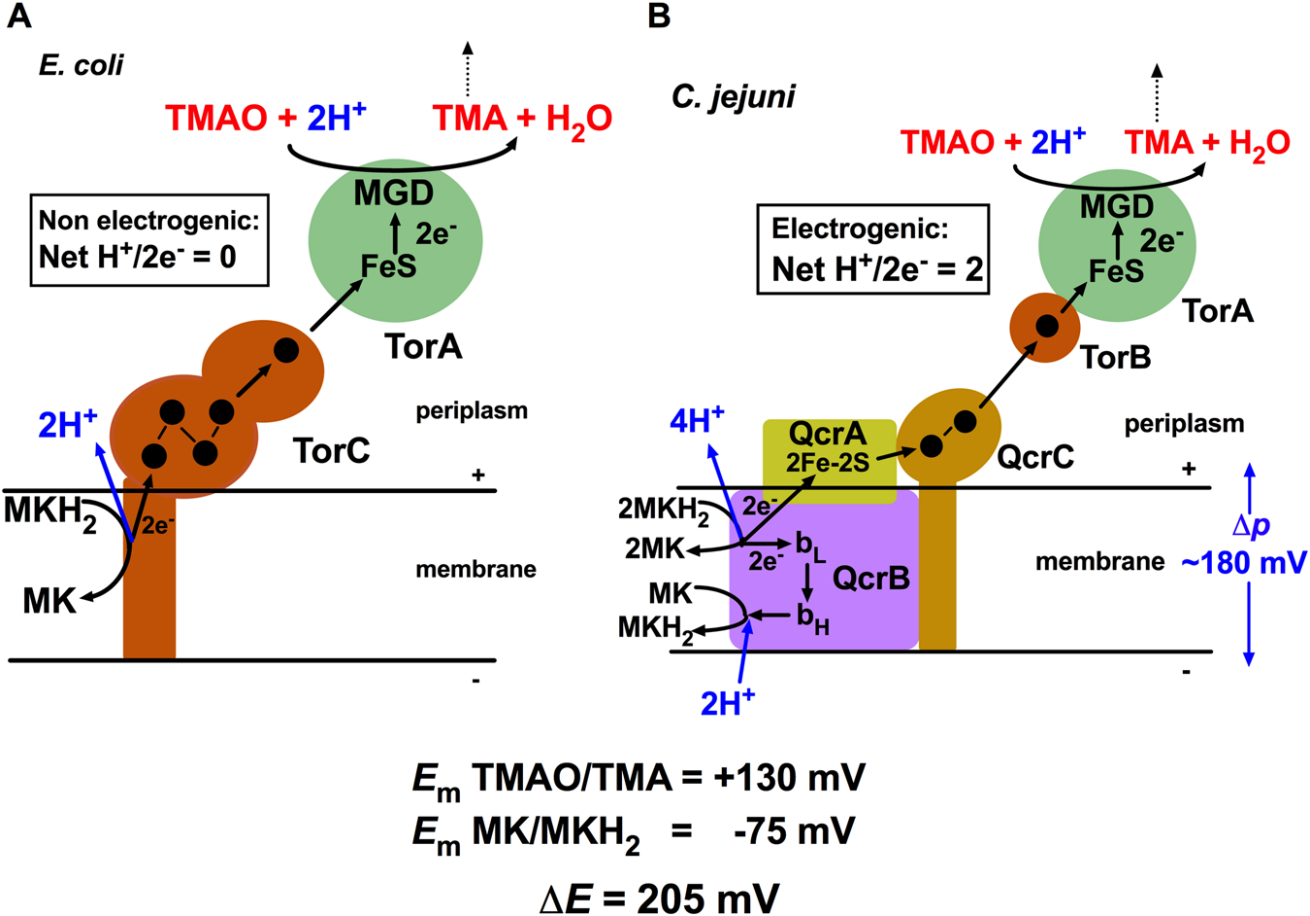
Comparison of the mechanism of TMAO reduction in *E*. *coli* and *C*. *jejuni*. (**A**) shows how quinol oxidation on the periplasmic side of the membrane in *E*. *coli* is coupled to electron transfer (black arrows) through the five haems in TorC (black filled circles) and the [4Fe-4S] and molybdopterin guanine dinucleotide (MGD) cofactors in TorA, ultimately reducing TMAO to TMA. Note that this mechanism is not energy-conserving (H^+^/2e^−^ transferred is 0) because proton release from quinol oxidation and proton uptake during TMAO reduction occur in the same compartment (the periplasm). (**B**) shows the proposed energy-conserving mechanism of TMAO reduction in *C*. *jejuni* coupled to the proton-motive Q cycle in the Qcr complex, which gives an overall net H^+^/2e^−^ ratio of 2. Although the redox span between menaquinol and TMAO is 205 mV, close to the typical value of ~180 mV for Δ*p*, continued diffusion of TMA away from the system (dashed black arrow) would shift the equilibrium and favour electrogenic TMAO reduction. TorB (Cj0265), homologous to the C-terminal domain of TorC (see Supplementary Fig. 2), most likely receives electrons directly from QcrC (black arrows), before transfer to the redox centres in TorA. b_L_, low-potential haem *b*; b_H_, high-potential haem *b* in the QcrB subunit.

That TMAO reduction is also Qcr dependent in *C*. *jejuni* now explains the long-standing puzzling observation that there is no homologue of the membrane bound pentahaem quinol dehydrogenase TorC in this bacterium^17,22^, and it has hitherto been unclear how quinol oxidation is coupled to TMAO reduction. TorC has been characterised best in *E*. *coli*^34^ and is a member of the widespread NapC /NrfH family, which are commonly tetrahaem proteins. It consists of an N-terminal membrane anchored domain, a central periplasmic domain containing the four core haem c centres and an additional C-terminal periplasmic domain containing a fifth *c*-type haem (Supplementary Fig. 2 and Fig. 7A). Electron transfer from TorC to the catalytic molybdoenzyme subunit TorA occurs via this fifth haem in the C-terminal domain^34^ (Fig. 7A). Interestingly, in *C*. *jejuni*, TorA (Cj0264) is associated with a small soluble monohaem *c*-type cytochrome (TorB; Cj0265), which is absent from *E*. *coli*, but which is homologous to the C-terminal domain of TorC (see the alignment in Supplementary Fig. 2 and Fig. 7B). Thus, we suggest that the functions of TorB and the C-terminal domain of TorC are equivalent and that this domain has been retained as a soluble protein in bacteria like *C*. *jejuni* that use a Qcr-dependent mechanism of TMAO reduction, presumably to specifically receive electrons from QcrC.

What are the bioenergetic implications of a Qcr-dependent mechanism of TMAO reduction? While it is clearly unfavourable thermodynamically for ubiquinol oxidation (*E*_*m*_ +90 mV) to drive the reduction of TMAO (*E*_m_ +130 mV) against a typical Δ*p* across the membrane of ~180 mV, TMAO reduction by menaquinol (*E*_m_ −75 mV) has a Δ*E* of 205 mV. Although this would seemingly be close to the limit of electrogenic function for a Qcr-dependent, Q-cycle mechanism across an energised membrane, the product of TMAO reduction is TMA, which is a water soluble gas which would tend to diffuse out of the system. This could help shift the equilibrium and provide a thermodynamic pull that would effectively increase the Δ*E* available to drive the reaction. It should be noted that *C*. *jejuni* also synthesises methylmenaquinone (MMK) with an *E*_m_ of −125 mV for the MMK/MMKH_2_ couple; if MMKH_2_ could be used to drive TMAO reduction then the Δ*E* increases to 255 mV. Thus, the model of TMAO reduction in *C*. *jejuni* we now propose (Fig. 7B) is, like nitrate reduction, electrogenic with a net H^+^/2e^−^ ratio from menaquinol oxidation of 2, whereas in *E*. *coli* it is 0, because TorC is a non proton-motive menaquinol dehydrogenase releasing protons into the periplasm (Fig. 7A); in the latter case, it is only at the level of the primary dehydrogenases that energy can be conserved during TMAO (or indeed Nap dependent nitrate) reduction.

We noted that ~2-fold more succinate was excreted during oxygen-limited growth of the *qcrABC* mutant on TMAO, which might be a compensatory mechanism to maintain redox balance by disposing of electrons through a Qcr independent route, i.e. fumarate reduction, although the low rate did not allow any significant growth. Both wild-type and mutant excreted some pyruvate under these oxygen-limited conditions, and this has been noted previously in *C*. *jejuni* grown under very low oxygen availability^35^.

Although we have focussed on the clear phenotypes of the *qcrABC* mutant in relation to nitrate and TMAO reduction under oxygen-limited growth conditions, we also noted that this mutant grew far more poorly with oxygen as electron acceptor compared to a mutant deficient in the cytochrome *c* oxidase, CcoNOQP. We hypothesised that a growth inhibiting build-up of ROS might result from removal of the Qcr complex, because of its role as electron donor to the two periplasmic cytochrome *c* peroxidases (Cj0020 and Cj0358). Measurements using a ROS sensitive dye suggested a much higher accumulation of ROS was indeed occurring in the *qcr* mutant compared to the wild-type. There may also be other reasons why ROS would accumulate in this mutant, for example if there is a reduction in its ability to properly oxidise the quinol pool and electrons are transferred to oxygen by non-physiological routes.

These new results concerning the reduction of nitrate and TMAO in *C*. *jejuni* not only change our overall view of the bioenergetics of this pathogen but, more generally, help to rationalise the use of menaquinol in electrogenic Qcr-dependent electron transport pathways to alternative acceptors of moderate redox potential. For example, the recent discovery of the TsdA type of bidirectional tetrathionate reductase in some strains of *C*. *jejuni* and many other bacteria^20^, which is a soluble periplasmic diheam cytochrome *c*, also suggests the involvement of the Qcr complex in this mode of tetrathionate reduction. The recent experimental determination of the midpoint redox potential of the tetrathionate/thiosulphate couple as +198 mV^36^ rather than the previously assumed value of +24 mV, means that the redox span using menaquinol to reduce tetrathionate is 273 mV, allowing the Qcr complex to operate electrogenically against the Δ*p*, as with TMAO and nitrate.

Nitrate, TMAO and tetrathionate are present in the mammalian host intestinal environment and there is evidence that *Salmonella* uses nitrate reduction via the Nap system^37^ and tetrathionate reduction (in this case via the molybdoenzyme Tttr^38^) to gain a competitive advantage in host infection. While *torA* deletion mutants of *C*. *jejuni* do not show gross colonisation defects in a chicken model of colonisation, a *napA* mutant colonised at lower levels than the wild-type^21^. Given the conservation of Qcr-dependent pathways to electron acceptors other than oxygen in many *C*. *jejuni* strains, we suggest they are important in the infection biology of this pathogen.

## Experimental Procedures

### Bacterial strains, media and general culture conditions

*Campylobacter jejuni* strain NCTC 11168 was the wild-type strain used in this study. An isogenic *ccoNOQP* deletion mutant was previously constructed and described^24^. Individual deletion mutants in *cccB* (*cj*1*020c::cat*) and *cccC* (*cj0037::kan*) have also been previously described and characterised^24^; a double mutant was made by transformation of the deletion mutant plasmid pGEM1020CAT^24^ into the *cccC* mutant. Construction of the *qcrABC* deletion mutant is described below. All strains were routinely grown on Columbia Blood Agar base CM0331 (Oxoid, Basingstoke, UK) containing 5% (v/v) lysed horse blood (SR0050C, Thermo Scientific) and 10 μg/ml each of amphotericin B and vancomycin (referred to as CBA media) at 42 °C under microaerobic conditions [10% (v/v) O_2_, 5% (v/v) CO_2_ and 85% (v/v) N_2_] in a MACS growth cabinet (Don Whitley Scientific, Shipley, UK). Selective antibiotics (either kanamycin or chloramphenicol) were added where appropriate to a final concentration of 50 μg ml^−1^. From plates, bacterial cells were inoculated in 50–100 ml Mueller-Hinton broth (Oxoid) plus 20mM L-Serine (MHS) in 250 ml conical flasks. Liquid cultures for microaerobic growth were shaken at 200 rpm on a KS125 IKA-labortechnic shaker (IKA, Staufen, Germany) at 42 °C in the MACS growth cabinet as above. For oxygen-limited growth, cells were grown in 200 ml MHS in 250 ml conical flasks in the MACS growth cabinet as above, but without shaking. Where required, the electron acceptors sodium fumarate, sodium nitrate or TMAO were added to a final concentration of 20 mM from filter-sterilised stocks.

### DNA manipulation and construction of a *qcrABC* deletion mutant

Chromosomal DNA of *C*. *jejuni* was extracted by using the wizard genomic DNA purification kit (Promega). Standard techniques were employed for the cloning, transformation, preparation and restriction analysis of plasmid DNA from *E*. *coli*^39^. A plasmid vector (pGEMQCR) for allelic exchange mutagenesis of *qcrABC* was assembled using pGEM3zf (Promega), 500 bp upstream and downstream fragments flanking *qcrABC* and a kanamycin resistance cassette derived from pJMK30^25^ using the Gibson isothermal assembly method, as previously described^24^. The two flanking fragments were PCR amplified using primers qcr_ISA_F1F/F1R and qcr_ISA_F2F/F2R (Table 1) with adapters homologous to 30 bp around the HincII site of the pGEM3zf multiple cloning site and 30 bp at the start or end of the kanamycin resistance cassette, which was amplified using primers Kan_F and Kan R (Table 1). An isothermal assembly reaction was carried out at 50 °C for 1 h with equimolar amounts of both flanking fragments, kanamycin cassette and HincII digested pGEM3zf in a reaction master mix containing isothermal assembly buffer, T5 Exonuclease, Phusion polymerase and Taq ligase^24^. The mixture was then transformed directly into competent *E*. *coli* DH5a cells with selection for kanamycin resistance. Plasmid pGEMQCR was checked by automated DNA sequencing using standard vector M13 primers. *C*. *jejuni* cells were grown in MHS overnight, pelleted and washed in 1 ml of ice cold wash buffer (9% (w/v) sucrose and 15% (w/v) glycerol in water), 3–4 times and finally resuspended in 200–300 μl of ice cold wash buffer. pGEMQCR was electrotransformed into these cells (2.5 kV, 200 Ohms, 25 μF; Bio-Rad Gene Pulser), which were then spread onto non-selective CBA plates. After overnight incubation, cell growth was transferrred to CBA plates containing kanamycin. Colonies appeared within 2–4 days, which were PCR screened with primers qcr_screen_F and qcr_screen_R (Table 1) that anneal approximately 100 bp upstream and downstream of the *qcr* operon respectively.

### RT-PCR

Aliquots (5 ml) of mid-log microaerobically grown cultures (OD600 nm ~0.6) were pelleted and washed in 1 ml 20 mM sodium phosphate buffer, pH 7.4, then 5 µl phenol and 50 µl ethanol mixed in before re-pelleting. Phenol-pellets were stored at −80 °C. RNA was purified from phenol-pellets using the TRIzol Max Bacterial RNA Isolation Kit (Thermo Fisher) and subsequently DNase treated using the TURBO DNase Kit (Invitrogen), following the manufacturers protocols. The absence of contaminating DNA was confirmed by PCR screening using MyTaq Red Mix (Bioline). RT-PCR was performed using the SensiFAST SYBR kit (Bioline), with 20 µl reactions contained in 96-well plates following the manufacturers recommendations. Genomic DNA serial dilution controls were performed for each primer set to generate a standard CT curve. The RT-PCR was performed in an Mx3005P cycler, controlled by MxPro software (Stratagene). CT values generated by the software were manually processed to generate fold RNA changes, relative to wildtype, using *gyrA* as the housekeeping control gene. DNA controls were performed in duplicate, while RNA screening reactions were performed in quadruplicate. Primers used for RT-PCR are listed in Table 1.

### Microaerobic and oxygen-limited growth curves, and determination of nitrite

For comparisons of microaerobic growth, cells from overnight starter cultures in the MACS cabinet were inoculated in 200 ml MHS in 500 ml conical flasks with appropriate antibiotics to a final OD (600 nm) of 0.1 and grown at 42 °C until an OD 600 nm of approximately 0.4–0.5. They were then back diluted to an OD 600 nm of 0.1 in fresh 100 ml MHS in shaken 250 ml conical flasks and the OD 600 nm monitored every hour using Jenway 6705 UV spectrophotometer. All Growth curves were done in triplicates. For growth and supernatant sample collection under oxygen-limited conditions, cells grown microaerobically as above, were back diluted to an OD of 0.1 in fresh 200 ml MHS in 250 ml conical flasks, with and without TMAO, nitrate or fumarate, and OD 600 nm monitored every hour. Cells in 1 ml samples were separated from the media supernatant by centrifugation (13,800 × g, 5 min) and the supernatants removed and stored frozen at −20 °C until ready for analysis. For nitrite determination, diluted culture supernatants (50 μl) from oxygen-limited growth experiments were added to 850 μl of 1% (w/v) sulphanilamide (Sigma) dissolved in 1 M HCl and 100 μl of 0.02% (w/v) naphthylethylenediamine (Sigma). After 15 min, the absorbance at 540 nm was measured and nitrite concentrations were determined by reference to a standard curve.

### ^1^H and ^13^C-NMR

For analysis of TMAO reduction, trimethylamine (TMA) concentrations in supernatant samples were quantified by ^1^H-NMR. To 800 μl of sample 10 μl of 100 mM trimethylsilyl propionate (TSP) was added as a 0 ppm chemical shift and quantitation reference. 450 μl of the mixture was transferred to NMR tubes, 50 μl of D_2_O was added and spectra acquired as described by Sellars *et al*.^17^. Integration of the single peak of TMA at 2.88–2.89 ppm and comparison with the TSP peak allowed TMA concentrations to be calculated. The ^13^C chemical shifts of succinate and pyruvate were obtained from a 2D ^1^H−^13^C HSQC spectrum, acquired with the standard Bruker pulse program, hsqcetgpsisp2, on an 800 MHz Avance I NMR spectrometer. The data were acquired with 2048 points in the direct dimension and 300 complex points in the indirect dimension, 64 transients per indirect point and a SW of 100 ppm for ^13^C.

### Total membrane protein isolation and detection of *c*-type cytochromes

Cell cultures were grown overnight in 500 ml MHS, harvested by centrifuging at 8,000 × g, 4 °C for 20 min and resuspended in 5 ml of 10 mM HEPES buffer (pH 7.4). Cells were broken by sonication for 6 × 20 s at a frequency of 16 microns amplitude (MSE sonicator). Unbroken cells and debris were removed by centrifuging at 15,000 × g, 4 °C for 30 min. The supernatant was then centrifuged at 100,000 × g, 4 °C for 1 h in a benchtop ultracentrifuge (Beckman). The supernatant was discarded and the membrane pellet was washed and resuspended in 1 ml 25 mM phosphate buffer (pH 7.4). Total protein concentration was determined by Lowry assay. Proteins were denatured gently by incubating for 1 h at 37 °C in SDS-PAGE sample buffer but without β-mercaptoethanol. Proteins were separated by SDS-PAGE on 10% acrylamide gels and either stained with Coomassie blue G250 or electroblotted onto nitrocellulose membrane (Hybond-C extra, GE Healthcare). Covalently bound haem was detected as haem-associated peroxidase activity^40^, using the enhanced chemiluminesence (ECL) kit from GE Healthcare. Images were obtained using a ChemiDoc XRS system (BioRad Inc) with an exposure time of 2 min.

### Measurement of substrate respiration rate in intact cells

Respiration rates were measured as the rate of oxygen consumption of cell suspensions in a Clark-type oxygen electrode using 10 mM sodium formate as electron donor, calibrated using air-saturated 25 mM phosphate buffer (pH 7.4) (200 nmol dissolved O_2_ ml^−1^ at 42 °C). Total protein concentration of the cell suspension was determined by Lowry assay at 600 nm and the specific rate of oxidation was calculated as nmol oxygen produced min^−1^ mg^−1^ total protein.

### Measurement of Reactive Oxygen Species (ROS)

Cells were grown microaerobically in MHS and harvested at mid-expoential growth phase by centrifugation (8,000 × g, for 3 min). Cell pellets were washed and resuspended in 5 ml of 25 mM phosphate buffer (pH 7.4). Cells were added to 6 ml of 25 mM phosphate buffer (pH 7.4) in 6-well plates to a final OD 600 nm of 0.2. 2′,7′ dihydrodichlorofluorescein diacetate (H2DCFDA; Life Technologies, USA), dissolved in 1% DMSO, was added to a final concentration of 10 μM at time zero and the plates incubated microaerobically at 42 °C. Samples (1 ml) were removed every 10 min and fluorescence emission at 538 nm measured on a Cary Eclipse (Agilent), fluorimeter, with excitation at 485 nm. Total protein concentration of the cell suspension was determined by Lowry assay and the data expressed as fluorescence intensity per mg protein.

## Electronic supplementary material

Supplementary information

## Data Availability

All data generated or analysed during this study are included in this published article.
