# Supplementary material for: Bacterial periplasmic nitrate and trimethylamine-N-oxide respiration coupled to menaquinol-cytochrome c reductase (Qcr): Implications for electrogenic reduction of alternative electron acceptors
Source: Sci Rep. 2018 Oct 19;8:15478. doi: 10.1038/s41598-018-33857-2 (PMC6195509; doi:10.1038/s41598-018-33857-2)
Supplement: Supplementary file 1 — Supplementary information [file 41598_2018_33857_MOESM1_ESM.pdf]

SUPPLEMENTARY INFORMATION

Bacterial periplasmic nitrate and trimethylamine-N-oxide respiration coupled to menaquinol-cytochrome *c* reductase (Qcr): Implications for electrogenic reduction of alternative electron acceptors

Nitanshu Garg, Aidan J. Taylor and David J. Kelly*

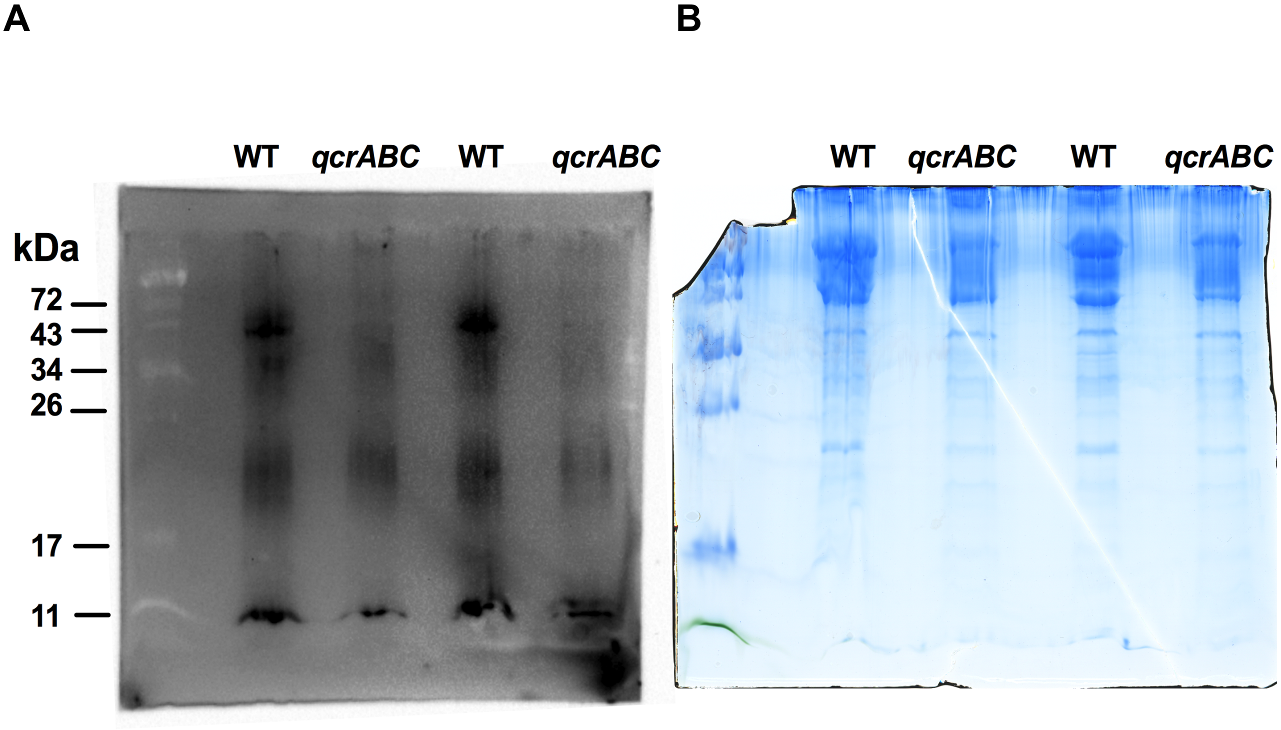

Supplementary Figure 1. Haem blot of *C. jejuni* wild-type and *qcrABC* mutant strains. (A) Full size, uncropped haem blot that Fig. 1C is derived from. (B) Coomassie blue stained SDS-PAGE gel of the same samples. Total membranes were prepared as described in Experimental Procedures and denatured gently by incubation at 37 °C for 1 h in Laemmli sample buffer without mercaptoethanol. 20 µg total protein was run per lane on two identical gels. One gel was electroblotted onto nitrocellulose membrane and stained for haem by enhanced chemiluminescence (Panel A). The Image was obtained using a ChemiDoc XRS system (BioRad Inc) with an exposure time of 2 min. The other gel was stained to show similar total protein loading in each lane (panel B). Panel (A) shows that a prominent haem stained band of ~ 42 kDa, the predicted size of QcrC, is missing in the *qcrABC* mutant.

```
                                                                    H1
           1
     TorC  MRKLWNALRRPSARWSVLALVAIGIVIGIALIVLPHVGIKVTSTTEFCVS
   Cj0265  ..................................................
Consensus  ..................................................

                                          H2
           51                                              100
     TorC  CHSMQPVYEEYKQSVHFQNASGVRAECHDCHIPPDIPGMVKRKLEASNDI
   Cj0265  ..................................................
Consensus  ..................................................

                                                     H3
           101                                             150
     TorC  YQTFIAHSIDTPEKFEAKRAELAEREWARMKENNSATCRSCHNYDAMDHA
   Cj0265  ..................................................
Consensus  ..................................................

                              H4
           151                                             200
     TorC  KQHPEAARQMKVAAKDNQSCIDCHKGIAHQLPDMSSGFRKQFDELRASAN
   Cj0265  .................................MKKIFISFV..VLATCL
Consensus  .................................MkkgFrkqf..lrAsan

           201                                             250
     TorC  DSGDTLYSIDIKPIYAAKGDKEASGSLLPASEVKVLKRDGDWLQIEITGW
   Cj0265  WAKNTAYTDEVVSLYLNKDDTKVIGRLLPTNPFEVLKSENNRVLLKIDGY
Consensus  dag#TaYsd#!kpiYaaKdDkeaiGrLLPanefeVLKr#n#rlqieIdGw

           251                                             300
     TorC  TESAGRQRVLTQFPGKRIFVASIRGDVQQQVKTLEKTTVADTNTEWSKLQ
   Cj0265  VNPKAPS.VIYFNDSQRIIVAAF....SKNTKLNFSQRITGKNGKWDKVS
Consensus  t#paarq.VitqndgqRIiVAai....qq#tKlnekqr!adkNgeWdKlq

                                          H5
           301                                             350
     TorC  ATAWMKKGDMVNDIKPIWAYADSLYNGTCNQCHGAPEIAHFDANGWIGTL
   Cj0265  LEIWADKKEFVKDNKEMLNRAKELFVNNCGICHAIHKEKEFTANAWPAIF
Consensus  aeaWadKg#mVnDnKeilarAdeL%nnnCnqCHaaheeaeFdANaWiail

           351                                   390
     TorC  NGMIGFTSLDKREERTLLKYLQMNASDTAGKAHGDKKEEK
   Cj0265  RSMADRTGIDKKDRWLVIEYLQKNAKDFKTK.........
Consensus  rgMadrTgiDKr#rrllieYLQkNAkDfagK.........
```

Supplementary Figure 2. Sequence alignment between TorC from *E. coli* K12 and Cj0265 (TorB) from *C. jejuni* NCTC 11168; H1-H5 (red) are the CXXCH C-haem ligation sites and the black boxed area in the N-terminal region of TorC shows the approximate position of the single transmembrane alpha-helix forming a membrane anchor. The C-terminal part of TorC containing the fifth C-haem ligation site is homologous over the entire length of Cj0265 and the single C-haem ligation site in Cj0265 is in the same relative position. Alignment was performed using MULTALIN with default parameters.
